# Disruption of the serine/threonine protein kinase H affects phthiocerol dimycocerosates synthesis in *Mycobacterium tuberculosis*

**DOI:** 10.1099/mic.0.062067-0

**Published:** 2013-04

**Authors:** Anaximandro Gómez-Velasco, Horacio Bach, Amrita K. Rana, Liam R. Cox, Apoorva Bhatt, Gurdyal S. Besra, Yossef Av-Gay

**Affiliations:** 1 Department of Medicine, Division of Infectious Diseases, Faculty of Medicine, University of British Columbia, Vancouver, BC, Canada; 2 School of Biosciences, University of Birmingham, Edgbaston, Birmingham B15 2TT, UK; 3 School of Chemistry, University of Birmingham, Edgbaston, Birmingham B15 2TT, UK

## Abstract

*Mycobacterium tuberculosis* possesses a complex cell wall that is unique and essential for interaction of the pathogen with its human host. Emerging evidence suggests that the biosynthesis of complex cell-wall lipids is mediated by serine/threonine protein kinases (STPKs). Herein, we show, using *in vivo* radiolabelling, MS and immunostaining analyses, that targeted deletion of one of the STPKs, *pknH,* attenuates the production of phthiocerol dimycocerosates (PDIMs), a major *M. tuberculosis* virulence lipid. Comparative protein expression analysis revealed that proteins in the PDIM biosynthetic pathway are differentially expressed in a deleted *pknH* strain. Furthermore, we analysed the composition of the major lipoglycans, lipoarabinomannan (LAM) and lipomannan (LM), and found a twofold higher LAM/LM ratio in the mutant strain. Thus, we provide experimental evidence that PknH contributes to the production and synthesis of *M. tuberculosis* cell-wall components.

## Introduction

**Fig. 1.**
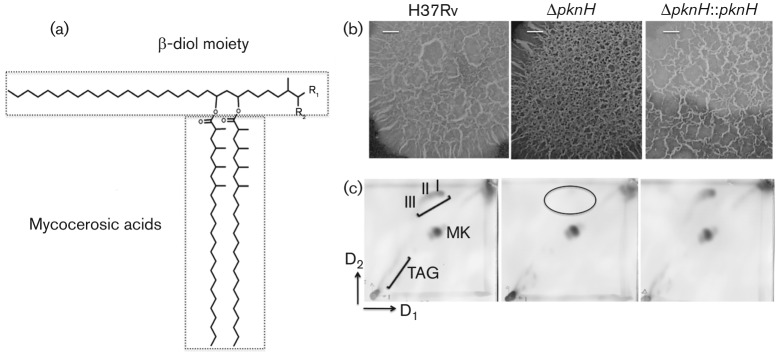
(a) PDIM structure. The long chain β-diol is esterified by multimethyl-branched fatty acids (mycocerosic phthioceranic acids). R_1_ is CH_2_-CH_3_ for phthiocerol series A and C or CH_3_ for phthiocerol series B; R_2_ is OCH_3_ for the phthiocerol family or is O for the phthiodiolone family (series C). (b) Colony morphology. Colonies of H37Rv wild-type, Δ*pknH* and Δ*pknH* : : *pknH* strains were grown on 7H10 agar plates supplemented with OADC. Colonies were obtained by inoculating 10 µl aliquots from cultures at OD_600_ 0.010. Plates were sealed and incubated at 37 °C for 3 weeks. Bars, 1 mm. (c) Apolar lipid profile of the Δ*pknH* mutant strain. Apolar lipid profile 2D-TLC reveals that PDIMs were not produced in the Δ*pknH* strain (circled), while the wild-type and the complemented strains produced similar amounts. PDIMs (I, phthiocerol series A; II, phthiocerol series B; III, series C in phthiodiolone family); MK, menaquinones; TAG, triacylglycerols.

*Mycobacterium tuberculosis* possesses a complex cell wall characterized by the presence of a high content and diverse array of lipids (Minnikin, 1982). Phthiocerol dimycocerosates (PDIMs) are a family of surface-exposed polyketide lipids which constitute the most-abundant free lipids found in the cell wall. These non-polar complex lipids are composed of a mixture of long chain β-diols (C_33_–C_41_) termed the phthiocerols, which, in turn, are esterified by two multimethyl-branched fatty acids, termed mycocerosic acids (Daffé & Laneelle, 1988; Minnikin *et al.*, 2002; Onwueme *et al.*, 2005). Depending on chemical modifications, the PDIMs are classified into series: the phthiocerol series A have a 3-methoxy group, the phthiocerol series B have a 2-methoxy group, whereas the series C, the phthiodiolone family, have a 2- or 3-keto group (Fig. 1a).

Biosynthesis, transport and translocation of PDIMs to the surface of the bacterium are well studied. Biosynthesis of PDIMs is initiated by activation and transfer of C_12_–C_18_ fatty acids by FadD26 (Trivedi *et al.*, 2004). The activated fatty acids are transferred to the type I polyketide synthases (PKSs) PpsA–PpsE, which elongate straight-chain fatty acids until the final phthiocerol backbone is synthesized (Azad *et al.*, 1997; Trivedi *et al.*, 2005). In parallel, an iterative type PKS, the Mas protein, produces mycocerosic acids, which, in turn, are transferred to the β-diol backbone of the phthiocerol by the acyltransferase PapA5 (Azad *et al.*, 1996; Onwueme *et al.*, 2004; Trivedi *et al.*, 2005). Release of the elongated phthiocerol moiety from PpsE is carried out by the type II thioesterase TesA (Alibaud *et al.*, 2011). Transport and translocation of PDIMs to the cell wall is carried out by either MmpL7 or DrrABC (Camacho *et al.*, 1999, 2001; Cox *et al.*, 1999).

Protein phosphorylation, carried out by protein kinases, is the principal mechanism by which extracellular environmental stimuli are translated into adaptive gene expression. The *M. tuberculosis* genome encodes 11 serine/threonine protein kinases (STPKs), shown to be involved in the regulation of pathogenesis, cell division and cell-wall synthesis (Av-Gay & Everett, 2000; Chao *et al.*, 2010a; Cole *et al.*, 1998). Recent studies have shown the involvement of two STPKs, PknB and PknD in the production of PDIMs. PknB was shown to phosphorylate PapA5 on threonine residues (Gupta *et al.*, 2009), whereas PknD may phosphorylate MmpL7 (Pérez *et al.*, 2006). These studies provide experimental evidence that PDIMs are regulated by STPKs.

We have previously shown that deletion of *pknH* leads to improved survival of the mutated strain in a BALB/c mouse model of infection, indicating that PknH is needed for *in vivo* bacterial growth (Papavinasasundaram *et al.*, 2005). More recently, we found that PknH is linked to the *M. tuberculosis* dormancy regulon by phosphorylating the control enzyme DosR (Chao *et al.*, 2010b). Previous studies have demonstrated that PknH is able to phosphorylate enzymes participating in cell-wall biosynthesis. *In vitro* kinase assays revealed that PknH phosphorylated EmbR, and this interaction may play a role in the transcription of the *embCAB* operon that encodes arabinosyltransferases (Molle *et al.*, 2003; Sharma *et al.*, 2006; Zheng *et al.*, 2007). Overexpression of PknH in *Mycobacterium smegmatis* activated EmbR, which induced the transcription of the *embCAB* operon, leading to a higher lipoarabinomannan (LAM) / lipomannan ( LM) ratio (Sharma *et al.*, 2006). *M. tuberculosis* PknH also phosphorylates DacB1, an enzyme that in *Bacillus subtilis* is a sporulation-specific protein involved in cell-wall biosynthesis (Zheng *et al.*, 2007). Together these results suggest that PknH plays an important role in the regulation of *M. tuberculosis* growth by controlling cell-wall compound synthesis and/or transport. Rationalizing that *M. tuberculosis* cell-wall components contribute to its virulence and that a Δ*pknH* deletion mutant strain has been shown to be hypervirulent (Papavinasasundaram *et al.*, 2005), we undertook a detailed cell-wall lipid analysis to investigate whether their biosyntheses were affected by PknH. In this study we show by radiolabelling, MS (lipidomics) and immunostaining analyses that PDIM production and the ratio of LAM to LM are specifically affected by knocking out the *pknH* gene.

## Methods

### 

#### Bacterial strains and growth conditions.

*M. tuberculosis* H37Rv, Δ*pknH* and Δ*pknH* : : *pknH* strains were used in this study (Papavinasasundaram *et al.*, 2005). Starter cultures were initiated from glycerol stocks using 10 ml 7H9 Middlebrook (Becton and Dickinson) broth supplemented with 10 % (v/v) oleic acid/albumin/dextrose/catalase (OADC), and 0.05 % (v/v) Tween-80 at 37 °C. Actively growing bacterial cells were used to start 500 ml cultures for rolling conditions (850 cm^3^ roller bottles; Greiner Bio-one, catalogue no. 680060, 1.25 r.p.m.) and were incubated at 37 °C. 7H11 agar medium (B&D) was prepared according to the manufacturer’s instructions and was supplemented with 10 % (v/v) OADC. Antibiotics were supplemented as required: 25 µg kanamycin ml^−1^ and 50 µg hygromycin ml^−1^.

#### Extraction of apolar and polar lipids.

For cell-wall lipid analysis, mycobacterial strains were grown at early exponential phase (OD_600_ ~1, measured by Optizen POP spectrophotometer, path length 10 mm). Cells were harvested by centrifugation, washed three times with PBS (100 mM K_2_HPO_4_, 10 mM NaCl, pH 7.4) and were autoclaved at 121 °C. Autoclaved cells were then lyophilized prior to extraction and purification of apolar and polar lipids as described previously (Besra, 1998; Dobson *et al.*, 1985). Each lipid extract (100 µg) was loaded onto TLC plates (silica gel 60F_254_, Merck) and was separated using 2D-TLC and solvent systems A–E (Besra, 1998; Dobson *et al.*, 1985). To visualize PDIMs, lipids were separated using a mixture of petroleum ether/ethyl acetate (98 : 2, v/v), three times in the first direction, while a mixture of petroleum ether/acetone (98 : 2, v/v) was used in the second direction. TLC plates were developed by staining with 5 % ethanolic phosphomolybdic acid, followed by charring at 100 °C.

For radiolabelling experiments [1-^14^C]-propionate 3.7×10^10^ Bq ml^−1^ [specific activity 54 mCi mmol^−1^ (1.998 GBq mmol^−1^); American Radiolabeled Chemicals] and [1,2-^14^C]-acetate 3.7×10^10^ Bq ml^−1^ [specific activity 57 mCi mmol^−1^ (2.109 GBq mmol^−1^)] were added at different time points (12 and 24 h, 5 and 10 days) to 10 ml mycobacterial cultures at 37 °C. PDIMs were extracted, their activities were measured in a scintillation counter (Beckman) and they were purified as above using preparative TLC. Equal radioactivity was loaded onto 2D-TLC. Autoradiograms were visualized using a Phosphorimager SI (Molecular Dynamics). For lipid quantification, spots were scraped from TLC plates and subjected to scintillation counting. For statistical analysis three independent biological replicates were used.

#### Fourier transform ion cyclotron resonance (FT-ICR) MS analysis.

Mycobacterial strains were grown as described above and total lipids were extracted using the Bligh–Dyer method (Bligh & Dyer, 1959). To avoid interference with the results Tween-80 used as a supplement in the culture was removed as described previously by Jain *et al.* (2007). Briefly, cell extracts were resuspended in a hexane/water mixture (50 : 50, v/v), mixed thoroughly and centrifuged at 3500 ***g*** for 5 min. The organic layer was extracted with water (five times). For lipidomic analysis, total lipids were resuspended in a chloroform/methanol mixture (2 : 1, v/v) and injected into an Apex-Oe 12-Tesla Hybrid quadrupole-FT-ICR machine (Bruker Daltonics), which was equipped with an Apollo electrospray ionization (ESI) ion source. Samples were infused into the MS instrument at a flow rate of 2 µl min^−1^ and were ionized with ESI. Mass spectra were acquired within a mass to charge (*m*/*z*) ratio range of 250–300 in either positive or negative mode, with broadband detection and using a data acquisition size of 1024 kilobytes per second. Each spectrum was accumulated from 100 scans. Total abundance of lipid species was calculated by summing the peak intensities as measured by FT-ICR, as reported previously by Jain *et al.* (2007).

#### Production of single-chain, fragment-variable (scFv) antibodies.

Purified PDIMs were kindly provided by Dr Jean-Marc Reyrat (INSERM-UMR, France). scFv antibodies against purified PDIMs were selected as described previously (Bach *et al.*, 2001). Briefly, 1.7 mg purified PDIMs was dissolved in 100 µl chloroform and then 100 µl 0.1 M 2-(*N*-morpholino)ethanesulfonic acid, pH 4.5, was added to the dissolved PDIMs. The sample was sonicated and mixed with 30 µl 10 nM stock solution of hydrazine. The mixture was incubated at 65 °C for 10 min. After cooling down the reaction to room temperature, the mixture was conjugated to 4 mg BSA using 1-ethyl-3-[3-dimethylaminopropyl] carbodiimide hydrochloride (Pierce), according to the manufacturer’s instructions. The Tomlinson I scFv antibodies library was kindly supplied by Geneservice, Cambridge, UK. The scFv antibodies were screened according to the instructions supplied with the library. PDIMs coupled to BSA were used as antigens for screening. Selected antibodies were subcloned into pMAL-C5X (NEB) and were produced as recombinant proteins fused to a maltose-binding protein (MBP) as described previously (Bach *et al.*, 2001).

#### Immunostaining and fluorescence microscopy.

Bacteria were labelled with rhodamine (10 mg ml^−1^) for 1 h at 37 °C with gentle rocking. Labelled bacteria were washed three times with PBS and three times with double-distilled water and were immobilized on coverslips by flaming. Coverslips containing bacterial cells were incubated with scFv antibodies at room temperature for 30 min, and unbound antibodies were washed away with PBS for 10 min (three times). Coverslips were further incubated with an anti-MBP antibody (1 : 1000 dilution) mixed with goat FITC-conjugated anti-mouse IgG secondary antibody (1 : 1000 dilution) at room temperature for 20 min. Coverslips were washed again with PBS for 10 min (three times) and mounted on glass slides containing FluorSave (Calbiochem). Samples were analysed by fluorescence microscopy as described previously by Sendide *et al.* (2004).

#### THP-1 cell infection and immunofluorescence microscopy.

The human monocyte cell line THP-1 (American Type Culture Collection) was cultured in RPMI 1640 (Sigma) supplemented with 1 % l-glutamine, 100 µg streptomycin ml^−1^, 100 U penicillin, 0.1 % fungizone (Invitrogen) and 10 % FCS (Sigma). THP-1 cells were seeded on coverslips at 5×10^6^ cells per well in 2 cm^2^ 24-well tissue culture plates and were differentiated by the addition of phorbol 12-myristate 13-acetate (20 ng ml^−1^) and they were incubated for 20 h in a humidified atmosphere (5 % CO_2_). Before infection, bacteria were labelled with rhodamine (10 mg ml^−1^) and were incubated with shaking for 30 min at 37 °C. After labelling, bacterial cells were washed with incomplete RPMI (supplemented only with FCS and l-glutamine) three times and were opsonized for 30 min at 37 °C with 10 % human serum. THP-1 cell monolayers were infected with bacteria at an m.o.i. of 10 : 1 (THP-1/bacteria) and were incubated at 37 °C and 5 % CO_2_ for 4 h. Non-internalized bacteria were removed by several washes with incomplete RPMI. Tissue cultures were further incubated at 37 °C and 5 % CO_2_ for 24 h.

Infected macrophages were fixed with 4 % *p*-formaldehyde for 30 min at room temperature and then permeabilized with saponin for 30 min. Coverslips were incubated with scFv antibodies at room temperature for 30 min, and any unbound antibody was washed away three times with PBS for 10 min. Coverslips were further incubated with anti-MBP (1 : 1000 dilution) and goat anti-mouse IgG-FITC conjugate secondary antibody (1 : 1000 dilution) at room temperature for 20 min. Coverslips were washed again three times with PBS for 10 min and were mounted on glass slides containing FluorSave (Calbiochem). Samples were analysed as described previously by Sendide *et al.* (2004).

#### Isobaric tags for relative and absolute quantitation (iTRAQ) analysis.

Proteomic analysis was performed as previously reported (Chao *et al.*, 2010b). Strains were grown in rolling cultures, harvested, washed and treated with 3 mM NaNO_2_. Total proteins were extracted, digested with trypsin at 37 °C overnight and then labelled with iTRAQ reagents. Tryptic-labelled peptides were separated using a polysulfethyl A [100×4.6 mm, 5 µM, 300 Å (30 nm)] strong cation exchange column (Poly LC) in the first dimension. The column was allowed to equilibrate for 20 min in buffer A [10 mM KH_2_PO_4_, pH 2.7 and 25 % acetonitrile (ACN)] before a gradient was applied; 0–35 % buffer B (10 mM KH_2_PO_4_, pH 2.7, 25 % ACN, 0.5 M KCl) for 30 min. The flow rate was set at 0.5 ml min^−1^. Tagged peptides were analysed by LC-MS/MS using an integrated Famos autosampler, Switchos II switching pump and Ultimate micropump system (LC Packings) with a hybrid Quadrupole-time of flight (TOF) LC-MS/MS mass spectrometer (QStar Pulsar i), equipped with a nanoelectrospray ionization source (Proxeon) and fitted with 10 µm fused-silica emitter tip (New Objective). The second dimensional chromatographic separation was carried out using a 75 µm×15 cm C_18_ PepMap Nano LC column [3 µm, 100 Å (10 nm); LC Packings] and a 300 µM×5 mm C_18_ PepMap 2 Guard column [5 µm, 100 Å (10 nm); LC Packings]. The mobile phase (solvent A) consisted of water/ACN (98 : 2, v:v) with 0.05 % formic acid for sample injection and equilibration on the guard column at a flow rate of 100 µl min^−1^. A linear gradient was created upon switching the tapping column inline by mixing with solvent B, which consisted of ACN/water (98 : 2, v:v) with 0.05 % formic acid, and the flow rate was reduced to 200 nl min^−1^ for high resolution chromatography and introduction into the mass spectrometer. MS data were acquired automatically using Analyst QS 1.0 software Service Pack 8 (ABI MDS SCIEX). An information-dependent acquisition method, consisting of a 1 s TOF MS survey scan of mass range 400–1200 atomic mass units (amu) and two 2.5 s product ion scans of mass range 100–1500 amu, was followed. The two most-intense peaks over 20 counts, with charge state 2–5, were selected for fragmentation, and a 6 amu window was used to prevent the peaks from the sample isotopic cluster from fragmenting again. MS/MS was put on an exclude list for 180 s. Curtain gas was at 23 °C, nitrogen was used as the collision gas and ionization tip voltage was 2700 V.

#### MS data analysis.

Data were obtained and analysed from two independent experiments. The identification and quantification of the proteins were performed using ProteinPilot 2.0.1 (Applied Biosystems/MDS Sciex). The Paragon algorithm integrated in the ProteinPilot software was used for peptide identification and was further processed by Pro Group algorithm for peptide identification and isoform-specific quantification, and the iTRAQ peak data were normalized for loading error by auto-biased corrections calculated using the ProteinPilot software. ProteinPilot software calculates an unused score of 2 for a peptide with a 99 % identity confidence and an unused score of 1.3 for a peptide with 95 % confidence level. An unused score of >2 indicates that a minimum of two peptides, one peptide with >95 % confidence plus at least one other peptide with less than 95 % confidence, were used exclusively for the identification of that protein. With a protein group of highly homologous proteins (identical peptides), peptides are arbitrarily assigned to one protein for which an unused score and iTRAQ ratio is determined. The percentage of protein covered by identified sequences at a 95% confidence level [% Cov(95)] is calculated by dividing the number of amino acids of peptides identified with 95 % confidence by the total number of amino acids in the protein. Relative quantification was performed on MS/MS scans and denotes the ratio of the areas under the peaks at 115 Da and 114 Da (untreated Δ*pknH*/WT), and 117 Da and 116 Da (nitrite-treated Δ*pknH*/WT).

#### Statistical analysis.

Statistical significance was determined with the unpaired two-tailed Student’s test with GraphPad Prism Version 5. *P*≤0.05 was considered statistically significant.

## Results

### The Δ*pknH* mutant produces low levels of PDIMs

*M. tuberculosis* virulence has been associated with a cording appearance and this is likely to be related to cell-wall components. In our previous study, we observed that the Δ*pknH* strain is hypervirulent in a mouse model (Papavinasasundaram *et al.*, 2005). As an initial analysis, we first characterized the colony morphology of all strains. An analysis of the parental wild-type and Δ*pknH* strains shows morphological differences in colony formations (Fig. 1b). While the cording appearance was similar for both the wild-type and the complemented strains, the Δ*pknH* shows a more ruffled shape (Fig. 1b). This appearance may have implications for the overall cell wall physical structure and therefore for *M. tuberculosis* physiology.

We next examined whether the deletion of *pknH* altered specific cell-wall components. For this purpose, we used a range of 2D-TLC solvent systems designed to systematically profile a wide range of mycobacterial lipids (Besra, 1998; Dobson *et al.*, 1985). Our screening demonstrated that the Δ*pknH* mutant failed to produce observable levels of PDIMs (Fig. 1c). 2D-TLC analyses of other cell-wall lipids show no apparent differences between the wild type and the Δ*pknH* strains (Fig. S1, available with the online version of this paper). We ruled out the possibility of polar effects associated with knock-outs of PDIM biosynthetic genes (Domenech & Reed, 2009) by showing that the complemented strain re-establishes the wild-type phenotype (Fig. 1c).

**Fig. 2.**
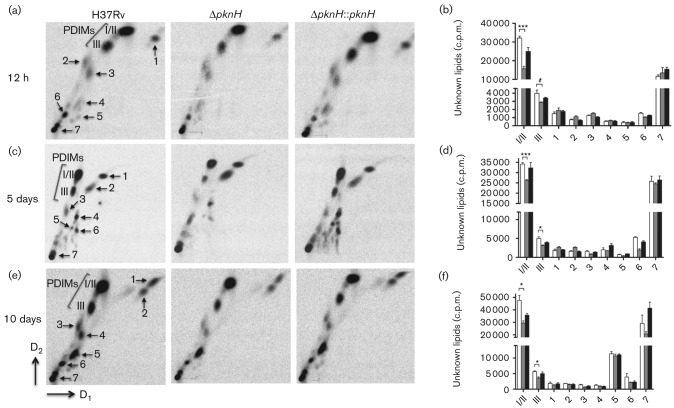
Production of PDIMs using propionate as a carbon source. Mycobacterial strains grown in 7H9 medium supplemented with OADC were labelled with [1-^14^C]-propionate and were further incubated at 37 °C to different time points. Apolar lipids were extracted as described in Methods. Equal amounts of lipid samples (100 000 c.p.m.) were loaded onto each TLC plate and plates were resolved as described in Methods. The observed general trend was the lower production of PDIMs in the mutant strain. Apolar lipid profiles for 12 and 24 h (a and b, respectively) were similar. However, levels and lipid profiles of unknown lipids changed at 5 days (c, d) and 10 days (e, f). Lipids were visualized by exposure to PhosphoImager SI. I, II and III, as described above. For lipid quantification spots from TLC plates, as shown in each 2D-TLC, were scraped and subjected to liquid scintillation counting. (b, d, f) Quantification of unknown lipids 1–7. Data are the means±sem from three independent biological experiments. *P≤0.05, ***P≤0.001, significant differences compared with wild-type samples by Student’s *t*-test. White bars, H37Rv; grey bars, Δ*pknH*; black bars, Δ*pknH *: : *pknH*.

To further confirm that preparative 2D-TLC demonstrates the absence of PDIMs, we labelled cell cultures with [1-C^14^]-propionate and [1,2-^14^C]-acetate and monitored their production at different time points (12 and 24 h, 5 and 10 days) (Figs. 2 and S2). Apolar lipids were further analysed by 2D-TLC. This sensitive technique showed that PDIMs were not fully abolished, but rather that their levels were reduced in the Δ*pknH* strain compared with those of the wild-type and the complemented strains (Figs 2 and S2). The growth of all strains was similar (data not shown), as reported previously (Papavinasasundaram *et al.*, 2005); therefore, the difference in PDIM biosynthesis was normalized by loading equal radioactivity (c.p.m.). Thus, the spots corresponding to PDIMs (series A/B and C, I/II and III in figures, respectively) from parental, Δ*pknH* and complemented strains were quantified by scintillation counting. For both radiolabelled carbon sources, the general trend observed was a reduction in PDIM production (Figs 2 and S2). The lipid profile of apolar lipids extracted from cells labelled with acetate remained similar at all time points (Fig. S2). Interestingly, the profile of apolar lipids from propionate-labelled cells remained similar at 12 and 24 h (Fig. 2a, b), but altered at 5 (Fig. 2c, d) and 10 days (Fig. 2e, f). Thus, radiolabelled-culture analysis, with both [1,2-^14^C]-acetate and [1-C^14^]-propionate, revealed a reduction of PDIM synthesis in the Δ*pknH* mutant as well as an alteration in the level and profile of other unidentified lipids.

### Structural analysis of total lipids from the Δ*pknH* strain

**Fig. 3.**
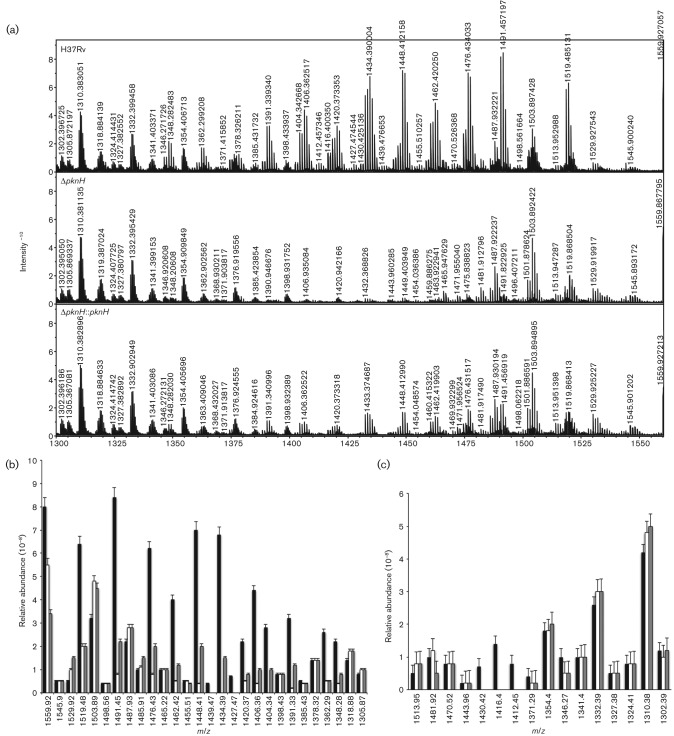
(a) PDIM region of FT-ICR mass spectra. Total lipids were extracted from mycobacterial strains by the Bligh–Dyer method. A series of molecular ions corresponding to PDIM masses *m*/*z* 1300–1600 were observed. Based on the relative intensity, PDIMs were more abundant in the wild-type and complemented strains compared with the Δ*pknH* strain. Absence of ion species in the region between 1390 and 1449 *m*/*z*, corresponding mainly to dimycocerosates A and B, was observed in the Δ*pknH* strain. Relative abundance of ion species corresponding to (b) dimycocerosates A/B and (c) dimycocerosates C. Abundance is shown as measured by FT-ICR. Black bars, H37Rv; white bars, Δ*pknH*; grey bars, Δ*pknH *: : *pknH*. Error bars represent sem.

Total lipids were extracted by the method described by Bligh & Dyer (1959) and were then subjected to FT-ICR MS analysis, shown previously to be a comprehensive analytical method to analyse complex lipids from *M. tuberculosis* (Jain *et al.*, 2007). The intensity of a series of molecular ions, corresponding to PDIM masses (C_86_–C_100_) in the *m*/*z* range 1300–1600, showed that these PDIMs species were more abundant in the parental wild-type strain than in the Δ*pknH* mutant (Fig. 3a). Comparison of the relative abundances of PDIM A–B and PDIM C lipid groups revealed significant reduction of both in the mutant strain (Fig. 3b, c). Complete absence of ion species in the 1390–1449 *m*/*z* region (DIM A/B) was observed in the mutant, while in the wild-type these peaks were more abundant, suggesting a higher production of these cell components (Fig. 3a and Table S1). The complemented strain partially restored the production of these same ion molecular species (Fig. 3a). We also observed that the ratio of total PDIM A/B to total DIM C lipids was 1.75 in the wild-type, which is similar to results in previous reports (Sartain *et al.*, 2011). Thus, this sensitive MS technique, together with the radiolabelled-lipid profile, confirmed that PDIMs are produced at lower levels in the Δ*pknH* strain compared with those of its parental strain.

### Immunofluorescent detection of PDIMs

**Fig. 4.**
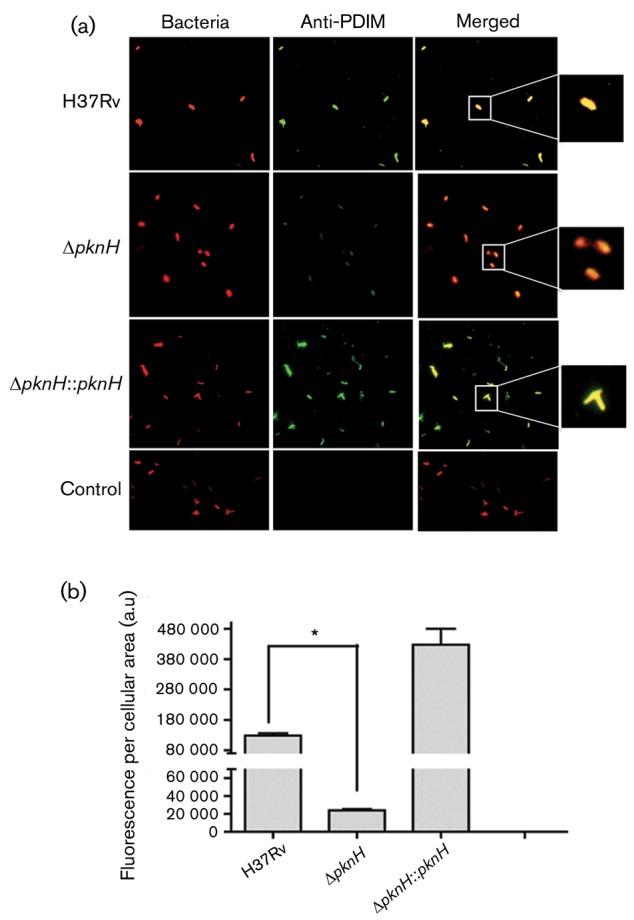
(a) Immunofluorescence microscopy analysis. Bacteria were labelled with rhodamine, and scFv antibodies against PDIMs were used as primary antibodies. Anti-MBP antibody coupled to goat anti-mouse IgG-FITC was used as the secondary antibody. The merged images are shown in the panels on the right. *M. smegmatis* was used as a negative control. (b) Immunofluorescence detection. Data represent the means±sd of green fluorescence intensity (labelled PDIMs) in arbitrary units (a.u.), which corresponds to labelled PDIMs, per cellular area; *n* = 50 single bacterial cells. *P≤0.05.

Both the MS- and TLC-based methods do not distinguish between cytosolic and cell-wall-bound lipids. In order to verify whether the observed PDIM levels reflect the relative abundance on the cell wall, we generated synthetic antibodies (scFv) against PDIMS and used them to monitor PDIM production using fluorescence microscopy in the examined strains. As a negative control, we incubated the antibodies with *M. smegmatis*, a mycobacterial strain known to be unable to produce PDIMs. Analysis of wild-type and Δ*pknH* : : *pknH* bacterial cells showed high-intensity labelling and 100 % co-localization for scFv antibodies against PDIMs in wild-type *M. tuberculosis* (Fig. 4a). A weak fluorescence signal was detected in the Δ*pknH* strain, corresponding to 18 % of the relative fluorescence compared with that the wild-type strain (Fig. 4b). Interestingly, higher fluorescence in the complemented strain was observed, suggesting that the overproduction of PDIMs may be related to uncontrolled expression of *pknH*.

We further examined PDIM production during *in vivo* analysis by infecting differentiated THP-1 cells with all strains, followed by immunostaining and microscopic analyses. As in our previous analysis, the wild-type and the Δ*pknH* : : *pknH* strains were strongly labelled on their surfaces with anti-PDIM antibodies (Fig. S3a), whereas in the Δ*pknH* strain only a portion of the fluorescence signal was detected (Fig. S3b).

### Proteins involved in PDIM synthesis are differentially regulated in the Δ*pknH* strain

We have previously analysed the proteome of the Δ*pknH* strain and compared it with that of the parental H37Rv strain. For this purpose, we used iTRAQ labelling of cultures grown under rolling conditions with or without the inducer nitric oxide in the form of 3 mM acidified nitrite (Chao *et al.*, 2010b). This proteomic approach has identified the role of PknH in the control of the mycobacterial dormancy regulon (Chao *et al.*, 2010b).

For the current study, we specifically examined the iTRAQ experiment data, focusing on proteins encoded by the PDIM biosynthetic pathway. We detected that, without induction, the levels of six out of the seven proteins participating in PDIM biosynthesis were similar when the mutant was compared with its parental strain. Interestingly, PpsE levels were higher in the Δ*pknH* strain compared with those in the parental strain (Table 1). PpsE levels remained high even in the presence of nitric oxide induction, a treatment which significantly induced the expression of only one biosynthetic protein, PpsD. Thus, the iTRAQ experiment reveals that protein levels of selected PDIM pathways are affected by PknH, which is is triggered by nitric oxide.

**Table 1.** iTRAQ analysis of PDIM biosynthetic proteins levels in Δ*pknH*/*pknH* with or without treatment with 3 mM acidified nitrite

Protein	ORF	Untreated ratio	Treated ratio (no. of peptides)
PpsA	Rv2931	1.05	0.93 (1)
PpsD	Rv2934	0.97	1.48 (1)
PpsE	Rv2935	2.36	1.93 (6)
PapA5	Rv2939	1.16	1.16 (1)
Mas	Rv2940c	1.20	0.89 (8)
FadD28	Rv2941	1.05	0.94 (7)
Ketoreductase	Rv2951c	1.06	1.11 (2)

### Alteration of LAM/LM ratio in the Δ*pknH* strain

Previous *in vitro* and *in vivo* studies have shown the interaction of PknH and EmbR (Molle *et al.*, 2003; Sharma *et al.*, 2006; Zheng *et al.*, 2007). Overexpression of PknH in *M. smegmatis* activates EmbR, which induces the transcription of the *embCAB* operon that encodes arabinosyltransferases, leading to a higher LAM/LM ratio (Sharma *et al.*, 2006). Therefore, we extended our study to *M. tuberculosis* to further analyse the content of these lipoglycans in the Δ*pknH* strain compared with those of the parental and complemented strains. LAM and LM were extracted, purified and analysed on 15 % SDS-PAGE, as shown in Fig. S4(a). The ratio of LAM to LM was significantly different between the wild-type and Δ*pknH* strains. This ratio was twofold higher in the mutant strain (Fig. S4b). The complemented strain showed a similar LAM/LM ratio to the parental strain (Fig. S4b). Although results agree with those from previously reported *M. smegmatis* studies (Sharma *et al.*, 2006), GC/MS analysis did not show any significant difference in arabinose and mannose content (Fig. S5). Nevertheless, we observed that the wild-type strain produced higher amounts of galactose and glucose compared with those of the mutant strain (Fig. S5).

## Discussion

Despite extensive knowledge of mycobacterial cell-wall biosynthesis, regulation of cell-wall components is still an emerging area of study. *In vitro* kinase studies suggest that STPKs are important regulatory enzymes in *M. tuberculosis* cell-wall biosynthesis (Chao *et al.*, 2010a). However, few studies have addressed *in vivo* interactions between STPKs and the biosynthetic enzymes of the cell wall.

We have shown previously that infection of BALB/c mice with a *pknH* knock-out strain resulted in a hypervirulent phenotype. These results suggest that PknH acts as an *in vivo* growth regulator (Papavinasasundaram *et al.*, 2005). In the present study, we carried out cell-wall lipid analyses to determine whether deletion of *pknH* affected cell-wall lipid biosynthesis. Strikingly, 2D-TLC analysis showed that PDIMs were not produced in the Δ*pknH* strain. However, when their production was evaluated using more sensitive techniques, such as radiolabelled 2D-TLC, MS and immunostaining, we found that although several species of PDIMs were produced, their overall levels were lower in the mutant compared with those in its parental wild-type or complemented strains. Additionally, we also observed the accumulation of unknown lipids in the Δ*pknH* strain.

The extracellular localization of PDIMs suggests an important role in cell-wall integrity and pathogenicity. *M. tuberculosis* strains unable to produce PDIMs or transport PDIMs to the cell wall are attenuated in animal models (Camacho *et al.*, 1999; Cox *et al.*, 1999; Kirksey *et al.*, 2011; Rousseau *et al.*, 2004; Yu *et al.*, 2012). However, a discrepancy has been found in two studies in which the H37Rv strain was used to assess the function of different genes (Ioerger *et al.*, 2010). These strains retained their virulence in mice despite harbouring a frameshift mutation in *mas* (Ioerger *et al.*, 2010), a gene encoding an enzyme that catalyses the synthesis of mycocerosic acids. These strains might have compensating mutations that retain virulence despite the loss of PDIMs (Ioerger *et al.*, 2010). It is still unknown how PDIMs mediate virulence, but a recent study has shown that PDIMs may facilitate a receptor-dependent phagocytosis and provide protection against phagosome acidification (Astarie-Dequeker *et al.*, 2009), although a precise molecular mechanism has not been defined.

Even though we found that PDIMs were produced at low levels in the mutant strain, we cannot exclude the possibility that biosynthesis of other cell-wall components or other signalling pathways might have been affected by disruption of the *pknH* gene. It is well known that PknH has effects on the transcription of the *embCAB* operon via phosphorylation of EmbR (Molle *et al.*, 2003; Zheng *et al.*, 2007). Furthermore, the overexpression of *pknH* in *M. smegmatis* results in a high LAM/LM ratio (Sharma *et al.*, 2006). Both LAM and LM act as ligands for host-cell receptors and contribute to the pathogenesis of *M. tuberculosis*, since they are located on its cell surface. It has been hypothesized that *M. tuberculosis* adapts to its human host by mimicking the glycoforms of mammalian mannoproteins (Torrelles & Schlesinger, 2010). Thus, the amount and nature of the mannose exposed on the surface might be major determinants for the phagocytosis and host response to *M. tuberculosis* (Torrelles & Schlesinger, 2010). Bacilli strains with reduced mannose are considered hypervirulent, whereas strains with abundant mannose on their surface have become more host adapted (Torrelles & Schlesinger, 2010). The latter strains may be highly successful in establishing an infection, potentially leading to a latent infection (Torrelles & Schlesinger, 2010). In line with this hypothesis, an unbalanced LAM/ LM ratio might partially explain the hypervirulence found in the Δ*pknH* strain. Indeed, our lipoglycan analysis revealed that the LAM/LM ratio was twofold higher in the Δ*pknH* strain compared with that in the wild-type, and this might be due to the transcriptional effect of the *embCAB* operon via EmbR phosphorylation. In fact, deletion of *pknH* from *M. tuberculosis* results in decreased transcription of *embB* and *embC* in cultures treated with sublethal doses of ethambutol (Papavinasasundaram *et al.*, 2005).

We also observed different production levels of unknown lipids (Fig. 2), which can also affect the course of *M. tuberculosis* pathogenesis. Diverse studies have demonstrated that correct structure and balance of cell-wall synthesis components have a marked effect on the pathogenesis of *M. tuberculosis.* For instance, a mutant lacking the *mmaA4* gene, which encodes the methyltransferase MmaA4 required for synthesis of keto- and methoxy-mycolic acids, displayed enhanced production of IL12p40, an important cytokine that controls intracellular infection (Dao *et al.*, 2008). Furthermore, the same study found that trehalose dimycolate (TDM), a modified mycolic acid linked with trehalose, derived from the Δ*mmaA4* mutant also stimulated IL12p40 (Dao *et al.*, 2008). The authors suggested that the different biological activities observed for the TDM wild-type and Δ*mmaA4* are based on the chemical and structural differences conferred by the functional groups of their mycolates (Dao *et al.*, 2008). Similarly, certain *M. tuberculosis* clinical isolates belonging to the W-Beijing family produce phenol glycolipids (PGLs) that are hypervirulent in murine disease models (Reed *et al.*, 2004). However, most *M. tuberculosis* strains, including the H37Rv strain, are devoid of PGLs. It has been proposed that *M. tuberculosis* strains are natural mutants deficient in PGLs due to a frameshift in the *pks15/1* gene (Constant *et al.*, 2002). Absence of this lipid in mutants lacking the *pks15/1* gene abrogates cytokine-repressing activity and leads to attenuation of virulence with extended survival in mouse infection studies (Constant *et al.*, 2002).

The current study leads us to propose that PknH positively regulates PDIM biosynthesis, as in the absence of *pknH* *M. tuberculosis* produces low levels of this class of lipids. Furthermore, our study also suggests that regulation of PDIM-biosynthetic proteins is fine-tuned rather than controlled through a strict on/off mechanism as proposed previously (Veyron-Churlet *et al.*, 2009). Recent studies have shown that phosphorylation of enzymes involved in mycolic acid biosynthesis results in a variety of biochemical outcomes. The enzymes involved in the FAS-II system during mycolic acid synthesis, such as malonyl-CoA : : AcpM transacylase (mtFabD) and the β-ketoacyl-AcpM synthases KasA and KasB, have been shown to undergo *in vitro* phosphorylation by different STPKs (Molle *et al.*, 2006). Interestingly, although KasA and KasB are similar enzymes that catalyse the condensation of acyl-AcpM and malonyl-AcpM (Kremer *et al.*, 2002; Schaeffer *et al.*, 2001), differential regulation by STPKs has been reported (Molle *et al.*, 2006). For instance, phosphorylation decreases the activity of KasA, while the enzymic activity of KasB is enhanced (Veyron-Churlet *et al.*, 2009). This differential effect of phosphorylation allows *M. tuberculosis* to produce immature mycolates by inhibiting KasA activity but enhancing KasB activity, which ensures the full-length mycolic acids required for bacilli virulence and intracellular survival (Veyron-Churlet *et al.*, 2009). A similar scenario may occur during PDIM biosynthesis in which different enzymes are phosphorylated by STPKs. Two studies have investigated the involvement of two STPKs in PDIM biosynthesis regulation. *In vitro* assays have shown that PknB is able to phosphorylate PapA5 on threonine residues and undergo reversible phosphorylation by Mstp (Gupta *et al.*, 2009), indicating that the transfer of mycocerosic acids onto the phthiocerol moiety is regulated by the PknB–Mstp protein complex. However, the *in vivo* effect of phosphorylation by PknB cannot be assessed since it is an essential gene product like PknA. Likewise, MmpL7, which is involved in PDIM transport, was found to be an endogenous substrate of PknD (Pérez *et al.*, 2006), suggesting that phosphorylation of MmpL7 can regulate deposition of PDIMs onto the cell wall.

The iTRAQ experiment has given insights into the nature of the signalling cascade mediated by PknH. Proteomic analysis revealed that seven proteins involved in PDIM synthesis are differentially regulated. Noteworthy is the level of PpsE expression which was maintained in untreated and treated cultures among the other proteins detected in the iTRAQ experiment. PpsE, a PKS, is the final enzyme involved in synthesis of the β-diol backbone PDIMs (Trivedi *et al.*, 2005). The upregulation of this enzyme suggests its importance in PDIM biosynthesis. In fact, the interaction of PpsE with TesA and MmpL7 has been shown (Jain & Cox, 2005; Rao & Ranganathan, 2004). The authors suggested that MmpL7 acts not only as a transporter but also as a scaffold to couple PDIM synthesis and transport (Jain & Cox, 2005). PpsE also interacts with the type II thioesterase TesA; the latter enzyme might be involved not only in releasing the growing product from PpsE, but also in housekeeping functions that remove inappropriate acyl units and/or aberrant acyl intermediates (Rao & Ranganathan, 2004). These data indicate that PpsE may act as an activator or inhibitor during final PDIM synthesis. On the other hand, the upregulation of PpsE in Δ*pknH* strains treated with nitric oxide might be due to the protein sensing the incorrect synthesis of PDIMs, because of the lack of signalling by PknH. Synthesis of PDIMs represents a high energy cost due to replication, transcription and translation of a gene cluster ~50 kbp. Thus, it is tempting to suggest that phosphorylation, mediated by STPKs and protein–protein complexes, may efficiently coordinate PDIM synthesis.

Further experiments are needed to address PDIM regulation via PknH phosphorylation; however, our study provides insights into the multiple *in vivo* signalling cascades that it employs. On the other hand, *M. tuberculosis* synthesizes and secretes diverse and complex lipids that interact with the host (Kremer & Besra, 2005). The outermost layer of the cell wall is composed of free lipids that include the trehalose ester family (sulfolipids, diacyltrehaloses, triacyltrehaloses and polyacyltrehaloses), the recently characterized mannosyl-β-1-phosphomycoketides, the phenolthiocerol and phthiocerol dimycocerosates, and the closely related phenolic glycolipids (Minnikin *et al.*, 2002). Therefore, based on this complexity, the study of particular lipids hinders *in vivo* lipid-specific studies, since there is not a unique and highly specific method to detect all *M. tuberculosis* cell-wall components. The development of scFv antibodies against PDIMs allowed us to monitor biosynthesis and localization at the bacterial cell surface. This new sensitive technique can specifically track a single-family lipid class; thus, it has potential for use in biochemical studies and studies of lipid biosynthesis during infection.
